# Flow Giese reaction using cyanoborohydride as a radical mediator

**DOI:** 10.3762/bjoc.9.208

**Published:** 2013-09-03

**Authors:** Takahide Fukuyama, Takuji Kawamoto, Mikako Kobayashi, Ilhyong Ryu

**Affiliations:** 1 Department of Chemistry, Graduate School of Science, Osaka Prefecture University, Sakai, Osaka 599-8531, Japan

**Keywords:** continuous flow system, cyanoborohydride, flow chemistry, iodoalkanes, microreactor, tin-free Giese reaction

## Abstract

Tin-free Giese reactions, employing primary, secondary, and tertiary alkyl iodides as radical precursors, ethyl acrylate as a radical trap, and sodium cyanoborohydride as a radical mediator, were examined in a continuous flow system. With the use of an automated flow microreactor, flow reaction conditions for the Giese reaction were quickly optimized, and it was found that a reaction temperature of 70 °C in combination with a residence time of 10–15 minutes gave good yields of the desired addition products.

## Introduction

**Scheme 1:**
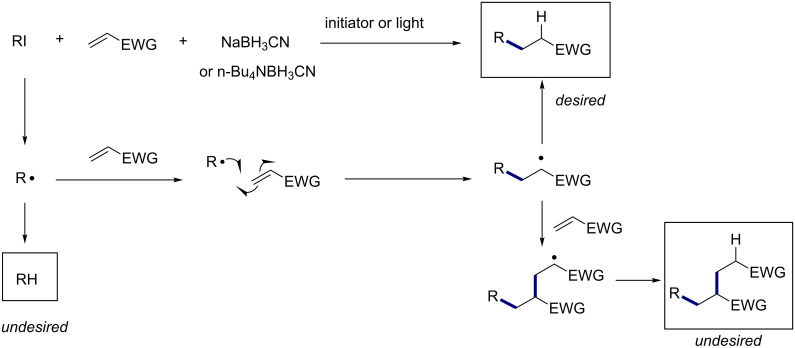
Giese reaction using borohydride-based radical mediators.

Organo halides are among the most useful precursors to access carbon radical species, and they have found numerous applications in chemical synthesis [1–5]. Alkyl radicals are classified as nucleophilic radicals, and therefore they are able to add preferentially to alkenes possessing an electron-withdrawing substituent [6–7]. This type of reductive radical addition reaction, better known as the Giese reaction, was historically carried out most by using tributyltin hydride as the radical mediator [8–9]. Recently borane derivatives such as borohydride reagents [10–13] or NHC-boranes [14–18] can be used in simple radical C–C bond forming reactions or radical reduction as efficient substitutes for tin hydride reagents, whose toxicity is of great concern to organic chemists. Thus far we have demonstrated the borohydride-based tin-free Giese reactions [10] and the related radical carbonylation and hydroxymethylation reaction [11–13,18] employing this methodology. In Scheme 1, a general mechanism of a borohydride-based Giese reaction with the possible products is shown.

In recent years, microreaction technologies have made a significant impact on chemical synthesis and production in terms of their advantageous characteristics, which include efficient mixing, efficient mass and heat transfer, and high operational safety [19–23]. Radical reactions also benefit from these advantages, and we have reported both photo- [24–26] and thermally-induced [27–30] radical reactions that are facilitated by flow reaction technology [31]. In this study, we report that cyanoborohydride-based Giese reactions of primary, secondary, and tertiary iodoalkanes with ethyl acrylate can be carried out efficiently using a microflow system. Optimal conditions for each substrate were quickly determined by the use of an automated microflow reactor [32], which revealed that running the continuous flow reactions at 70 °C for 10–15 min gave good yields of Giese addition products with effective suppression of the byproducts.

## Results and Discussion

**Figure 1:**
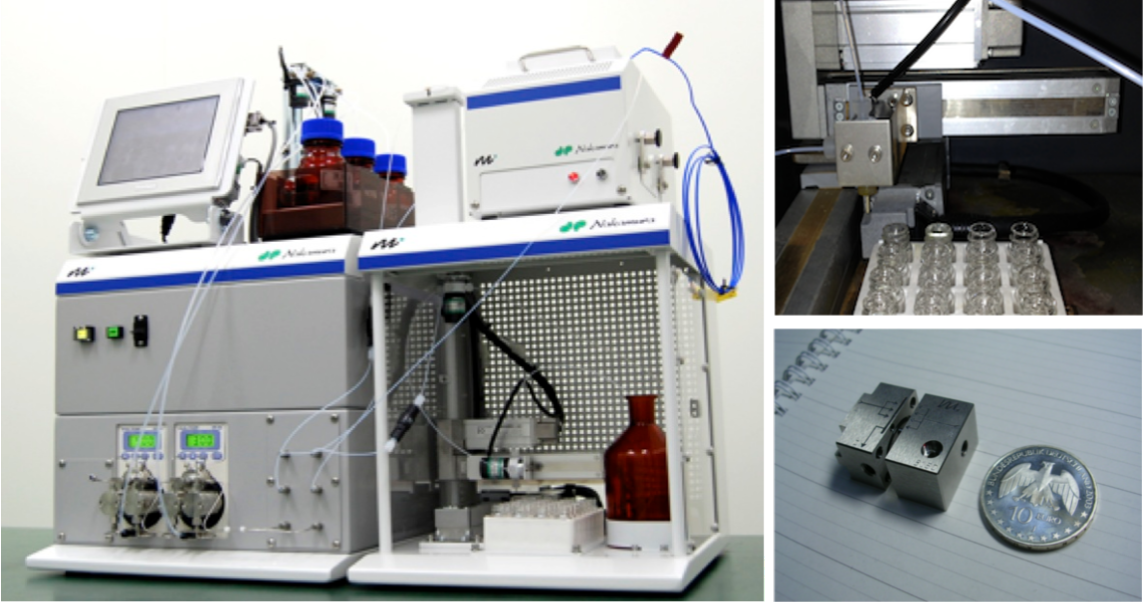
Pictures of the flow microreactor system (MiChS® System X-1), a micromixer (MiChS β-150, channel width: 150 μm), and a fraction collector used for this study.

We employed an automated microflow reactor system, MiChS^®^ System X-1 [33], equipped with a fraction collector, which allows screening of up to 20 reaction conditions in one operation through the programming of temperature and flow rates (Figure 1).

**Scheme 2:**
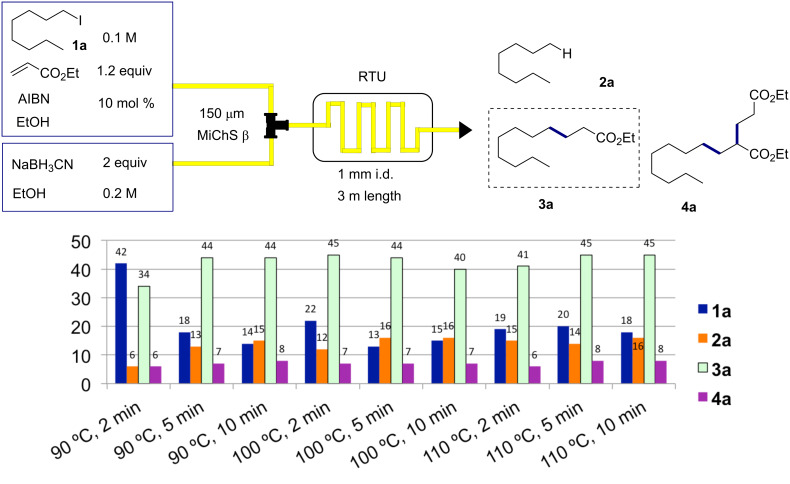
First screening for the reaction of **1a** at different temperatures (90–110 °C) and residence times (2–10 min) in the presence of AIBN.

Initially, the reaction of 1-iodooctane (**1a**) with ethyl acrylate in the presence of NaBH_3_CN (2 equiv) and 10 mol % AIBN (2,2’-azobisisobutyronitrile) was investigated. A variety of different temperatures (90–110 °C) and residence times (2–10 min) were screened. The reaction of **1a** with ethyl acrylate was found to give the desired Giese reaction product **3a** together with two main byproducts, octane (**2a**) and the 1:2 addition adduct **4a**. As shown in Scheme 2, higher reaction temperatures tended to result in the formation of increased amounts of octane (**2a**). Under the same reaction conditions, the radical mediator Bu_4_NBH_3_CN gave similar results, whereas the reaction with Bu_4_NBH_4_ was found not to be suitable, since the competing reduction leading to **2a** became the dominant product from the reaction.

**Scheme 3:**
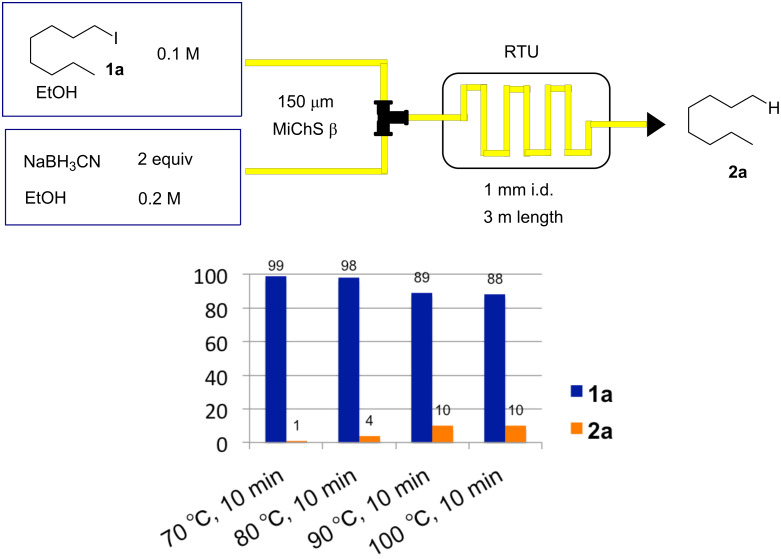
Background reduction of **1a** with NaBH_3_CN.

To check the background hydride reduction of **1a** with NaBH_3_CN, we treated **1a** with 2 equiv of NaBH_3_CN at various temperatures (70–100 °C) for 10 min in the absence of a radical initiator and ethyl acrylate (Scheme 3). The reduction product **2a** was not formed in large amounts and we found that its formation was effectively suppressed by lowering the temperature to 70 °C.

**Scheme 4:**
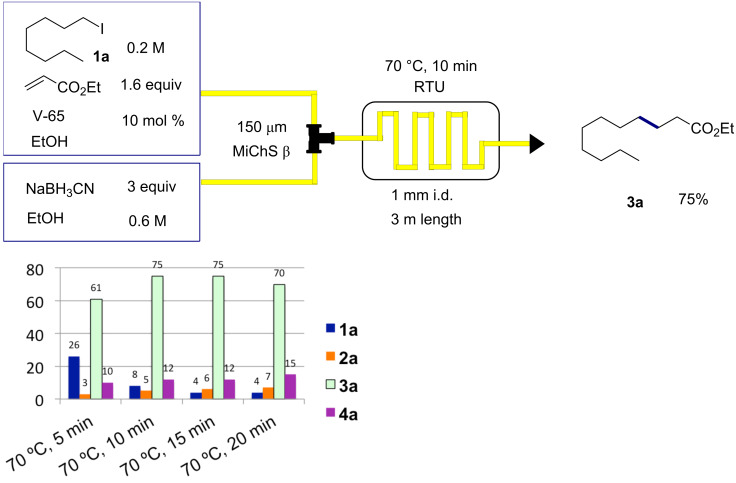
Second screening at 70 °C and residence time (5–20 min) in the presence of V-65.

**Figure 2:**
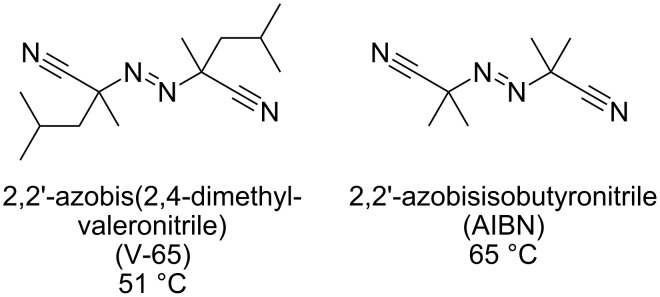
Structures of V-65 and AIBN and their ten hour half-life decomposition temperature.

Setting the reaction temperature to 70 °C, we then further optimize the other reaction conditions. Consequently we found that the desired Giese product **3a** could be obtained in 75% yield (Scheme 4) when the reaction was carried out with 1.6 equiv of ethyl acrylate and 3 equiv of NaBH_3_CN and 10 min residence time in the presence of V-65 (2,2’-azobis(2,4-dimethylvaleronitrile)) as the radical initiator, which decomposes at a lower temperature than AIBN (Figure 2). For comparison, we also carried out a batch reaction using a 20 mL test tube on 0.5 mmol scale under similar reaction conditions (70 °C (bath temp.), 10 min), which gave only 34% yield of **3a** and a large amount of recovered **1a**. We assume that excellent thermal efficiency inherent to tiny reaction channels would ensure efficient reaction in the microreactors.

**Scheme 5:**
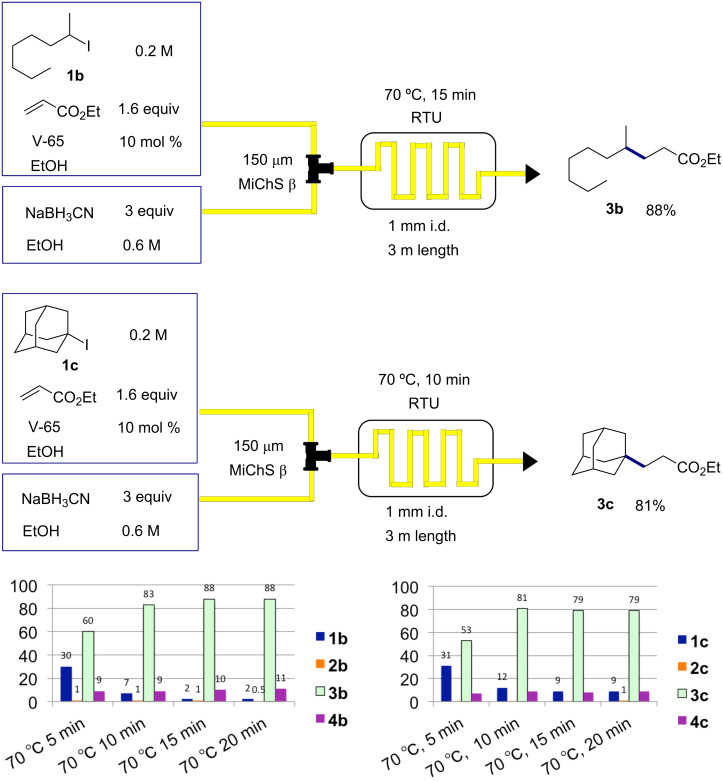
Cyanoborohydride mediated Giese reaction of **1b** and **1c** with ethyl acrylate.

We then carried out the optimization of the reaction conditions for the secondary and tertiary alkyl iodides, 2-iodooctane (**1b**) and 1-iodoadamantane (**1c**), reacting with ethyl acrylate. We were pleased to find that under similar reaction conditions (70 °C, 10–15 min) these two flow Giese reactions worked well to give the corresponding addition products **3b** and **3c** in 88 and 81% yield, respectively (Scheme 5). It should be noted that for these secondary and tertiary substrates, simple reduction to give octane (**2b)** or adamantane (**2c)** was hardly observed.

## Conclusion

The cyanoborohydride-mediated Giese reaction of alkyl iodides **1a**, **1b**, and **1c** with ethyl acrylate was studied in a continuous microflow reaction system. Optimized conditions with minimum formation of byproducts for the conversion of **1a** to **3a** were rapidly located by the use of an automated microflow system, MiChS^®^ X-1, equipped with a static mixer having 150 μm width and an automated fraction collector. Using the optimized flow conditions (70 °C, 10–15 min), high yielding conversions of **1b** to **3b** and **1c** to **3c** were also obtained.

## Supporting Information

File 1 Typical experimental procedure and supplementary experimental data.
