# Supplementary material for: Flow Giese reaction using cyanoborohydride as a radical mediator
Source: Beilstein J Org Chem. 2013 Sep 3;9:1791–6. doi: 10.3762/bjoc.9.208 (PMC3778382; doi:10.3762/bjoc.9.208)
Supplement: File 1 — Typical experimental procedure and supplementary experimental data. [file Beilstein_J_Org_Chem-09-1791-s001.pdf]

# Supporting Information

**for**

# Flow Giese reaction using cyanoborohydride as a radical mediator

Takahide Fukuyama, Takuji Kawamoto, Mikako Kobayashi and Ilhyong Ryu*

Address: Department of Chemistry, Graduate School of Science, Osaka Prefecture University, Sakai, Osaka 599-8531, Japan

Email: Ilhyong Ryu* - ryu@c.s.osakafu-u.ac.jp

* Corresponding author

## Typical experimental procedure and supplementary experimental data

**Table of Contents**

General remarks S2

Experimental procedure S2

Additional experiments S3

**General remarks**

The reactions performed using an automated microflow reactor system, MiChS® System X-1, equipped with a micromixer (MiChS β-150, 150 μm), a residence time unit (1 mm i.d. and 3 m length), two dual plunger HPLC pumps and a fraction collector, which allows screening of up to 20 reaction conditions in one operation through the programming of temperature and flow rates. Gas chromatography analysis was performed with an Shimazu GC-2014$AF_{SC}$ under the following conditions: initial oven temp. 60 °C, hold at this temperature for 5 min, first ramp 20 °C/min to 250 °C, hold at this temperature for 5 min. All GC yields were determined using decane as an internal standard. Response factors were calculated by the GC analysis of a mixture of authentic samples and an internal standard. $Bu_4NBH_4$ and $Bu_4NBH_3CN$ were prepared in a similar manner as described for corresponding borohydride[1]. Compounds **1b**[2] and **1c**[2] were prepared according to the literature procedures. Other reagents were purchased commercially and used without further purification.

**Typical procedure for tin-free Giese reaction in a microflow system (Scheme 3).**

A 50 mL EtOH solution of **1a** (10 mmol, 2.4 g, 0.2 M), ethyl acrylate (16 mmol, 1.6 g, 1.6 equiv), V-65 (1 mmol, 248.3 mg, 10 mol %), and decane as an internal standard (400 mg) (solution A), and a 50 mL EtOH solution of $NaBH_3CN$ (30 mmol, 1.87 g, 3 equiv) (solution B) were prepared and placed in bottles A and B respectively. These two bottles were connected by PTFE tube to the automated microflow system, MiChS® system X-1, equipped with a micromixer (MiChS β-150, 150 μm), a residence time unit (RTU, 1 mm i.d. and 3 m length) and an automated fraction collector. Four different conditions (residence time = 5, 10, 15, and 20 min at 70 °C, relative flow rate: A:B = 1:1) were inputted through a touch panel and the program was run. The reaction mixtures for each condition were sampled (1 mL) automatically in vials containing 1 mL of $H_2O$ by a fraction collector. Products were extracted with $Et_2O$ (1 mL), and the yields were determined by GC analysis equipped with a flame ionization detector.

[1] Hutchins, R. O.; Kandasamy, D. *J. Am. Chem. Soc*. **1973**, *95*, 6131.

[2] Olah, G. A.; Narang, S. C.; Gupta, B. G. B.; Malhotra, R. *J. Org. Chem*. **1979**, *44*, 1247.

## Additional Experiments:

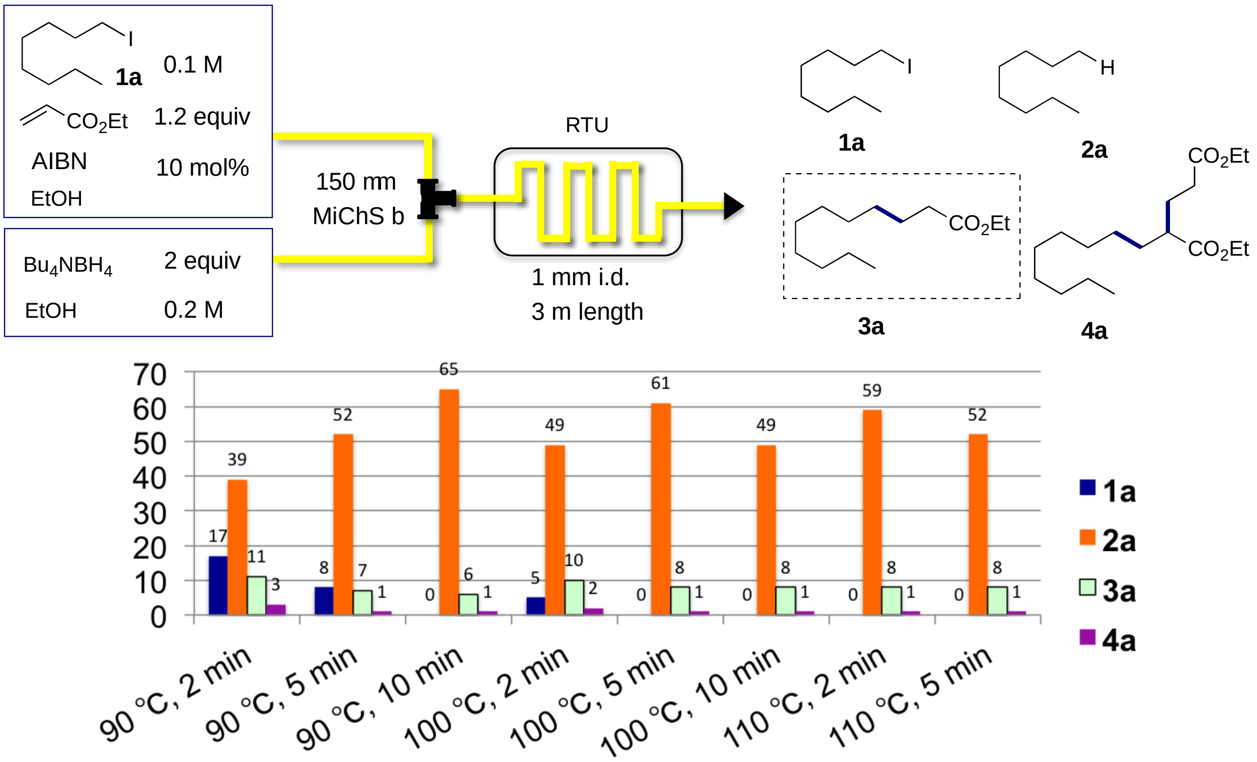


**Scheme S1:** Screening for the reaction of **1a** with $Bu_4NBH_4$ at different temperatures (90–110 °C) and residence times (2–10 min) in the presence of AIBN.

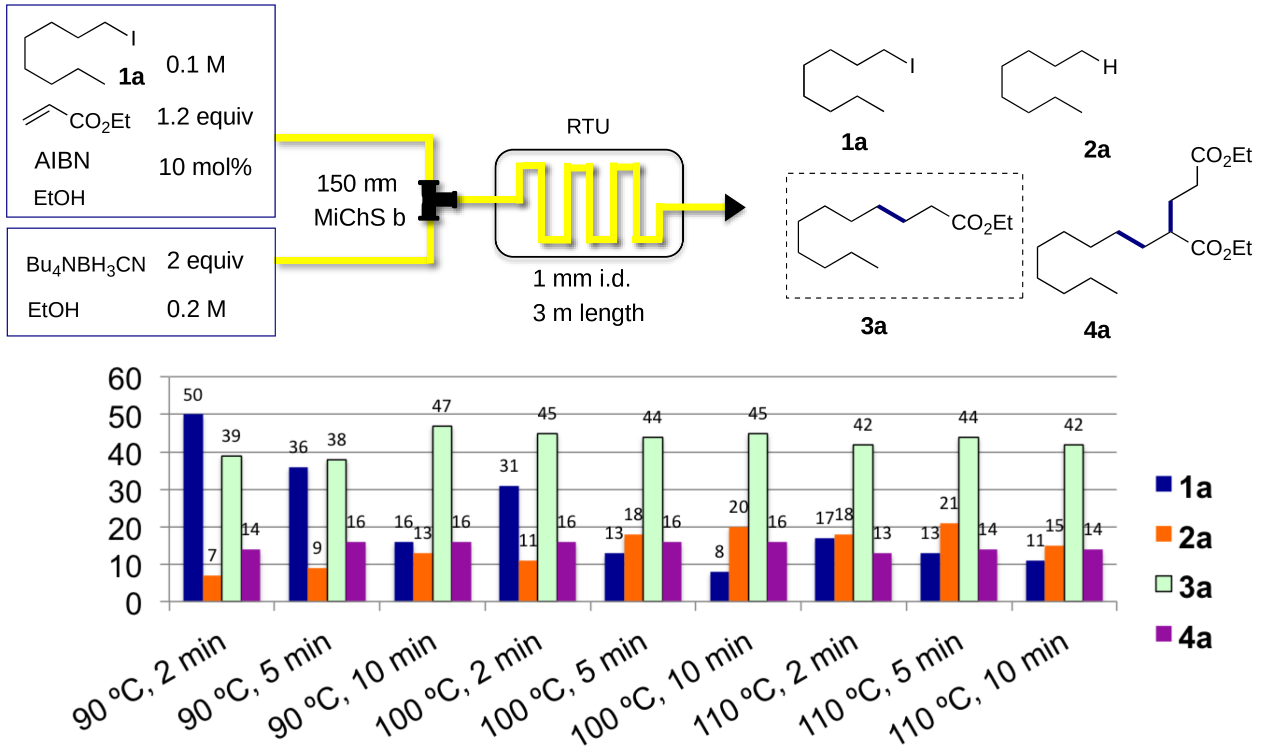


**Scheme S2:** Screening for the reaction of **1a** with $Bu_4NBH_3CN$ at different temperatures (90–110 °C) and residence times (2–10 min) in the presence of AIBN.
